# Novel Chlorhexidine-Loaded Polymeric Nanoparticles for Root Canal Treatment

**DOI:** 10.3390/jfb9020029

**Published:** 2018-04-17

**Authors:** Gina Quiram, Francisco Montagner, Kelli L. Palmer, Mihaela C. Stefan, Katherine E. Washington, Danieli C. Rodrigues

**Affiliations:** 1 Department of Bioengineering, University of Texas at Dallas, 800 W Campbell, Richardson, TX 75080, USA; Gina.Quiram@utdallas.edu (G.Q.); mihaela@utdallas.edu (M.C.S.); 2 Department of Conservative Dentistry, Dental School, Federal University of Rio Grande do Sul, Rua Ramiro Barcelos 2492, 90460-001 Porto Alegre, Brazil; francisco.montagner@ufrgs.br; 3 Department of Biological Sciences University of Texas at Dallas, 800 W Campbell, Richardson, TX 75080, USA; Kelli.Palmer@utdallas.edu; 4 Department of Chemistry, University of Texas at Dallas, 800 W Campbell, Richardson, TX 75080, USA; katherine.washington@utdallas.edu

**Keywords:** chlorhexidine, dentin permeability, dentin tubules, drug encapsulation, polymeric nanoparticles, poly(ethylene glycol)-*b*-poly(lactic acid)

## Abstract

Persistence of microorganisms in dentinal tubules after root canal chemo-mechanical preparation has been well documented. The complex anatomy of the root canal and dentinal buffering ability make delivery of antimicrobial agents difficult. This work explores the use of a novel trilayered nanoparticle (TNP) drug delivery system that encapsulates chlorhexidine digluconate, which is aimed at improving the disinfection of the root canal system. Chlorhexidine digluconate was encapsulated inside polymeric self-assembled TNPs. These were self-assembled through water-in-oil emulsion from poly(ethylene glycol)-*b*-poly(lactic acid) (PEG-*b*-PLA), a di-block copolymer, with one hydrophilic segment and another hydrophobic. The resulting TNPs were physicochemically characterized and their antimicrobial effectiveness was evaluated against *Enterococcus faecalis* using a broth inhibition method. The hydrophilic interior of the TNPs successfully entrapped chlorhexidine digluconate. The resulting TNPs had particle size ranging from 140–295 nm, with adequate encapsulation efficiency, and maintained inhibition of bacteria over 21 days. The delivery of antibacterial irrigants throughout the dentinal matrix by employing the TNP system described in this work may be an effective alternative to improve root canal disinfection.

## 1. Introduction

The translation of controlled drug delivery technologies in dentistry, especially in Endodontics, has been somewhat limited. The applications of nanoparticles as vehicles for drug delivery during root canal treatment procedures have been particularly concentrated in the blending of nanocomposites, such as zinc oxide, into endodontic sealers or root filling assemblies [1]. Recently, Shrestha and Kishen (2016) revised the current application of nanoparticles in Endodontics and reported that they were tested as solutions for irrigation, medication and as additive within sealers/restorative materials [2]. Nevertheless, further studies on drug release from loaded nanoparticles have not been tested as part of the presently intracanal medicament systems that are used to promote the reduction of residual microorganisms. An extensive variety of drugs can be conveyed through a number of avenues using degradable nanoparticles [3,4]. These nanocarriers can be utilized to carry and distribute hydrophilic and hydrophobic drugs, and other biological macromolecules [5,6,7,8,9,10]. Nanoparticles can thus be tailored to transport different drugs that are important in root canal treatment, such as chlorhexidine (CHX).

Chlorhexidine (CHX) is widely used in dentistry due to its broad-spectrum and potent antimicrobial action [11]. It is a strong antiseptic bisguanide with a di-cationic structure (positively charged) at pH levels above 3.5 that has bactericidal and bacteriostatic effects [12,13]. Furthermore, CHX demonstrates substantivity properties, which permits it to reside attached to different tissues and provide a long-lasting antibacterial activity of 8 to 12 h [14]. CHX has been used previously in two different forms, hydrogel or liquid, to create formulations that are designed to accompany chemo-mechanical preparation, as a mean of reducing the bacterial load within the complex root canal system. CHX has been proved highly effective as an intracanal treatment alone or combined with calcium hydroxide. Gomes et al. (2003), in an in vitro study, observed that chlorhexidine gel completely inhibited the growth of *Enterococcus faecalis* after 1, 2, 7, and 15 days [15]. Furthermore, Rosenthal et al. (2004) showed that bovine root dentine can retain CHX and can release it in concentrations that can inhibit *E. faecalis* for up to 12 weeks [16]. Siqueira et al. (2007) demonstrated that chemomechanical preparation with 0.12% CHX solution significantly reduced the number of intracanal bacteria in teeth from patients with primary intraradicular infections and chronic apical periodontitis [17]. However, it failed to render the canal free of cultivable bacteria in 50% of the cases. The application of a 7-day intracanal dressing with Ca(OH)_2_/CHX paste increased significantly the number of cases yielding negative cultures.

To improve bacterial reduction in infected root canal systems and to generate an environment that is optimal for periapical healing, it is required to prolong the time release of CHX beyond the current delivery systems currently available. Nanoencapsulation could offer a better medicament release control while further lengthening the delivery period of the bactericidal in dentinal tissues [18]. Thus, the aim of this study is to develop an innovative approach to extend the antimicrobial action of CHX by encapsulating the drug in poly(ethylene glycol)-*b*-poly(lactic acid) (PEG-*b*-PLA) nanoparticles. Poly(lactide) (PLA) and its derivatives are the most widely used polymers for the self-assembly of synthetic nanoparticles [19]. These polymers are known for both their biocompatibility, resorbability through natural physiological pathways, and their ability to transport a diverse range of drugs that can be lipophilic, hydrophilic, or amphiphilic [20]. Nanoparticle size distribution and encapsulation efficiency are significantly correlated to the molecular weight of the polymer and the ratio used of drug-to-polymer during self-assembly [21].

Thus, deploying nanoparticles that are specifically designed to encapsulate the drug, is expected to allow for the delivery of the bactericidal for a prolonged period of time and in a continuous manner, which will directly target the dentinal matrix. Prior work by Haseeb et al. demonstrated that poly(ethylene glycol)-*block*-poly(lactic acid) (PEG-*b*-PLA) bilayer nanoparticles (BNPs) for CHX encapsulation can be effectively self-assembled to enhance drug bioavailability and target drug distribution to the complex dentinal matrix [22]. The previous study demonstrated that self-assembly of these nanoparticles achieved sustained inhibition of bacteria, which could potentially be promoted for use in the treatment of root canal systems. The BNPs presented a hydrophilic corona with a hydrophobic interior space that encapsulated CHX. However, the synthesis of this kind of nanoparticle was experimentally difficult to reproduce due to the low solubility of CHX in water (800 mg/L) and in most solvents [22,23].

This study aimed to improve the physiochemical properties of poly(ethylene glycol)-*b*-poly(lactic acid) (PEG-*b*-PLA) nanoparticles through self-assembly of a trilayer nanoparticles (TNPs) system that were loaded with chlorhexidine digluconate (CHX-digluconate), a stable salt form of chlorhexidine (CHX). It was hypothesized that TNPs would provide prolonged drug release of up to 21 days, improving the antimicrobial effect of intracanal bactericidals. The TNPs encapsulated hydrophilic drugs that produced nanoparticles with a relatively packed structure in the range of 140–295 nm. In this study, TNPs encapsulated with CHX-digluconate were chemically and physically characterized and their antimicrobial efficiency estimated.

## 2. Results

### 2.1. Molecular Weight and Ratio of Polymeric Chains Estimation

**Figure 1:**
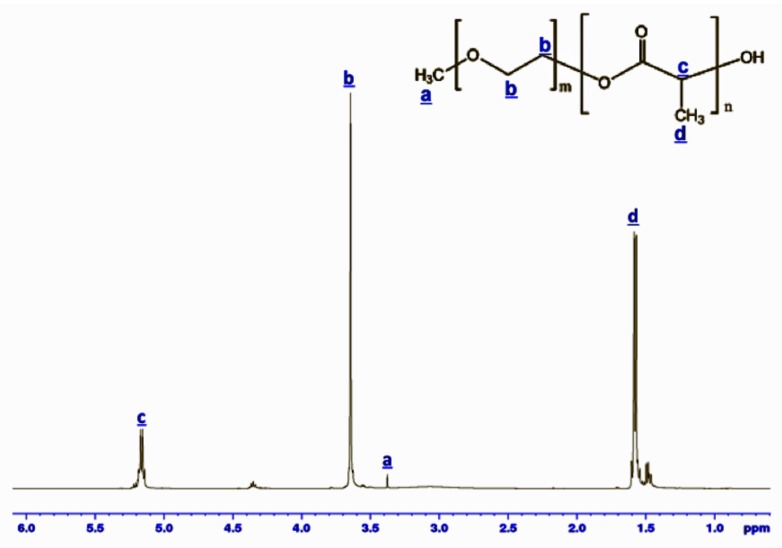
^1^H NMR spectra of the synthesized poly(ethylene glycol)-*b*-poly(L-lactide) block copolymer.

Synthesized PEG-*b*-PLA block copolymer was analyzed by ^1^H NMR. The PEG-*b*-PLA block copolymer number-average molecular weight was 4690 Daltons and the structure configuration of the block copolymer was 43 mol % PEG and 57 mol % PLA (Figure 1). The peaks at 3.64 ppm and 3.38 ppm corresponded to methylene units and CH_3_O– in the PEG block, respectively. Signals at 1.47 ppm and 5.16 ppm are attributed to the hydrogen atoms in CH_3_– and CH– groups from PLA segments, respectively [24].

### 2.2. Size Distribution of Reverse Nanoparticles

The water-in-oil emulsion formulation was studied to assess the physicochemical equilibrium and suitable size distribution of the subsequent reverse nanoparticles (RNPs) in the presence of CHX-digluconate. Size distribution of RNPs that were assembled in 1,3-dimethyl-2-imidazolidinone (DMI) and N,N-dimethylacetoamide (DMA) had an average size diameter of 164 nm and a polydispersity index (PDI) of 0.33. These outcomes are means of three independent consecutive measurements. 

The water-in-oil emulsion prepared with the organic solvents DMI and DMA, which density had been adjusted to 1.00 g/cm^3^, was stable even after 24 h. There was no phase separation, and the size distribution of the RNPs as determined through DLS analysis remained roughly the same.

### 2.3. Size Distribution and Morphology of Trilayered Nanoparticles

**Figure 2:**
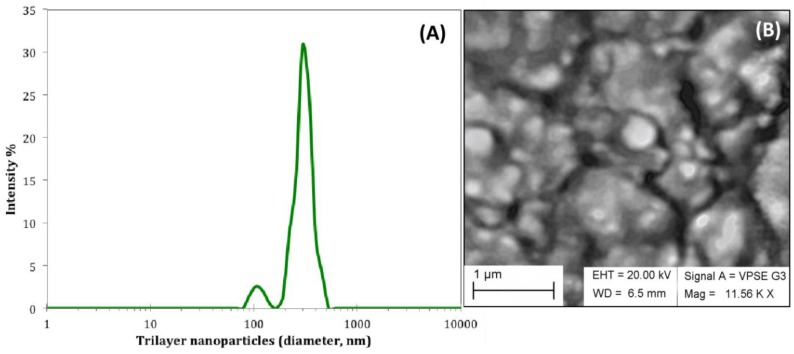
(**A**) Average DLS frequency distribution of trilayered nanoparticles (TNPs) presented in logarithmic scale as a function of intensity (*n* = 4), which encapsulated CHX-digluconate in the most inner core. (**B**) The SEM image shows TNPs after the freeze-drying process necessary to avoid diffusion of CHX and erosion of the polymeric nanoparticle. SEM imaging was done in variable pressure aperture.

The water-in-oil emulsion that contained RNPs was added to an aqueous solution containing the PEG-*b*-PLA block copolymer to create the trilayered nanoparticles (TNPs). Figure 2A shows the average size distribution of TNPs with one predominant peak at 295 nm that corresponded to the TNPs while the less intense peak at 105 nm could be related to the formation of BNPs as byproduct. This experiment was replicated four times (*n* = 4) and each time the DLS frequency spectra was evaluated.

The inner core of the conventional BNP is hydrophobic, while these TNPs contain a hydrophilic core that encapsulates CHX-digluconate, which is a hydrophilic drug. The shell of the TNP was composed of the overlapping regions of the block copolymer that corresponded to its hydrophobic part from PLA.

Energy dispersive X-ray spectroscopy (EDS) elemental analysis, in variable pressure (VP) mode, confirmed the presence of CHX-digluconate in the encapsulated nanoparticles by signal emission of chlorine (Cl) (Table 1).

**Figure 3:**
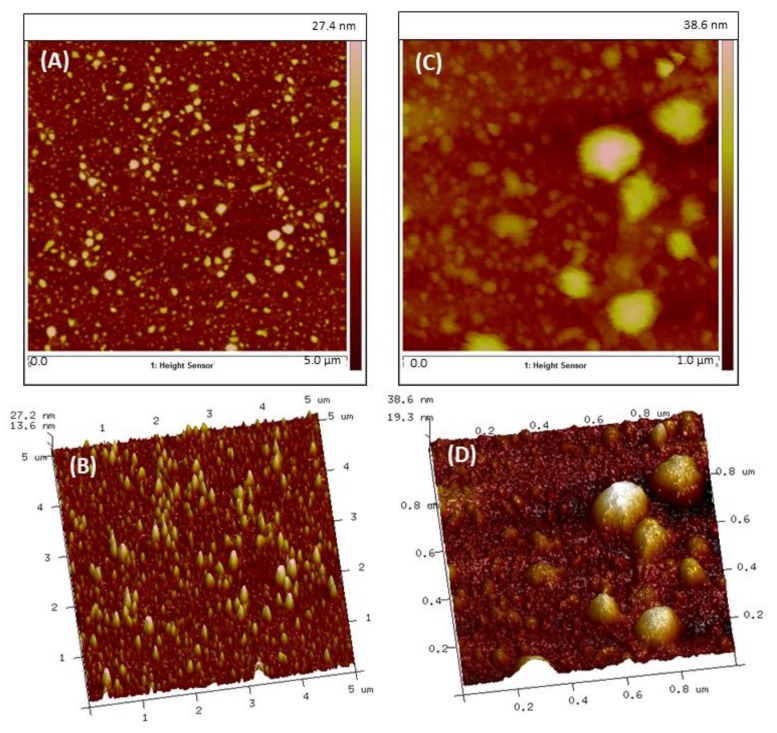
AFM analysis in air PeakForceTM QNM mode images of TNPs assembled from RNPs. (**A**) 2D (**B**) 3D topographic images of 5.0 × 5.0 μm scan. (**C**) 2D (**D**) 3D topographic images of 1.0 × 1.0 μm scan.

As with most investigative methodologies, it is frequently best to use a combination of procedures to characterize nanoparticles. 3D and 2D atomic force microscopy (AFM) imaging of TNPs that were purified through dialysis was also used to assess the morphological structure of TNPs (Figure 3). PeakForce^TM^ QNM (quantitative nanomechanics) data from AFM revealed information about sample surface profiles and particle analysis defined features of well-isolated nanoparticles. From this analysis, the average diameter range of TNPs was 140–245 nm, although there was also a significant number of BNPs in the diameter range of 40 to 100 nm. In general, scanning electron microscopy (SEM) images and AFM analyses presented here were consistent with DLS data.

Examination of the morphological mapping and quantitative measurements were made with section analysis and verified with particle analysis. Section analysis revealed information about sample surface contours and particle investigation demarcated structures of well-isolated particles or features of interest. From this study, the median diameter range of TNPs was 140–245 nm, nonetheless there was also a substantial number of other nanoparticles in the diameter range of 40 to 100 nm, which could be credited to the formation of BNPs as a byproduct. In general, SEM images and AFM analyses presented here were consistent with DLS data.

### 2.4. Nanoparticle Loading Efficiency

The self-assembly of TNPs proved to be a reproducible method to encapsulate CHX-digluconate for controlled release of CHX-digluconate. Investigation of the amount of drug encapsulated in TNPs was determined using UV-Vis spectroscopy at the wavelength at which CHX-digluconate has its maximum absorbance, λ_max_ = 281 nm. The solutions used to plot this calibration curve ranged from 0.064 mg/mL to 0.64 mg/mL, with a correlation coefficient R^2^ = 0.995.

From the absorbance analyses of the CHX loaded TNPs at 281 nm and using Equations (1)–(3) (as described in Materials and Methods), the drug encapsulation efficiency (DEE) was calculated, in relation to the theoretical drug loading (TDL), and the experimental drug loading (EDL). The self-assembly method employed here allowed for a drug encapsulation efficiency (DEE) of 84.5%, with TDL of 3.8% and EDL of 3.2%. This was the result for a PEG-*b*-PLA to CHX-digluconate ratio of 4 to 1 to make RNPs. For the second polymeric layer that makes the TNPs the amount of PEG-*b*-PLA necessary was 2.5 times higher than the one used for RNPs.

### 2.5. Antimicrobial Effectiveness

**Figure 4:**
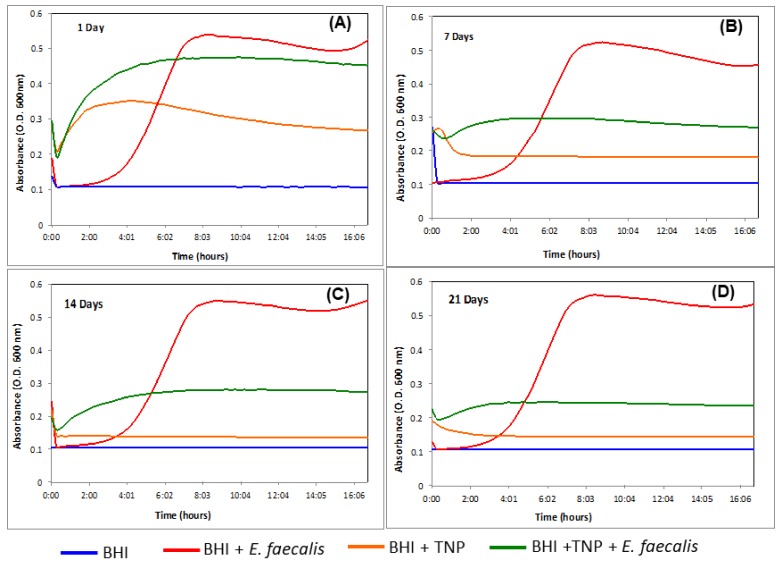
The effect CHX released from TNPs had on *E. faecalis*. TNPs were immersed in brain heart infusion (BHI) for (**A**) 1 day, (**B**) 7 days, (**C**) 14 days, and (**D**) 21 days, then tested against *E. faecalis*. Optical density (O.D.) measured at 600 nm. The OD data was used to generate bacterial growth curves made by averaging triplicates from three trials together, not including clear outliers obtained during the experiments.

The TNPs drug release was tested in vitro by submerging them in brain heart infusion (BHI) broth at 37 °C, to simulate the body’s physiological temperature. CHX-digluconate TNPs were immersed in BHI for varied periods (1 day, 7 days, 14 days, and 21 days). Then these broths were inoculated with *E. faecalis* and their optical density (OD) data recorded over 17 h. This data was used to generate bacterial growth curves made by averaging triplicates from three trials together. Representative results from the bacterial proliferation assays are shown in Figure 4 for 1 day, 7 days, 14 days and 21 days.

The drug release profile showed a biphasic pattern with a rapid initial burst release followed by a sustained release of CHX-digluconate. After TNPs were incubated for 1 day there was a 14% bacterial inhibition, after 7 days of incubation 50% bacterial inhibition, after 14 days of incubation 63% bacterial inhibition, and 80% bacterial inhibition after 21 incubation days.

The OD data at the intermediate time period, when the control was entering the growth phase, was also analyzed using statistical comparisons. A one-way ANOVA was conducted at 95% significance level. When the growth curves of the broth that had contained the TNPs (BHI + TNP + *E. faecalis)* were compared to the control (BHI + *E. faecalis*), a significant difference was observed (*p* ≤ 0.05). The results also indicated that the nanoparticle remained effective for the period investigated (up to 21 days).

## 3. Discussion

This work investigated the self-assembly, physicochemical characterization, and antimicrobial effectiveness of an advanced nanoparticle engineering technology to be delivered inside root canal systems. The aim of adapting a new controlled release nanoparticle system was to achieve sustained delivery of CHX-digluconate throughout the complex root canal for up to 21 days. The initial hypothesis was that nanoparticles self-assemble with a proper core/shell (PEG/PLA) ratio to improve CHX-digluconate encapsulation efficacy, with a narrow size distribution, and an efficient release profile of CHX-digluconate loaded polymeric nanoparticles. This TNP formulation improved the physicochemical equilibrium of the nanoparticles without the use of additional surfactants in the aqueous mixture solution.

**Figure 5:**
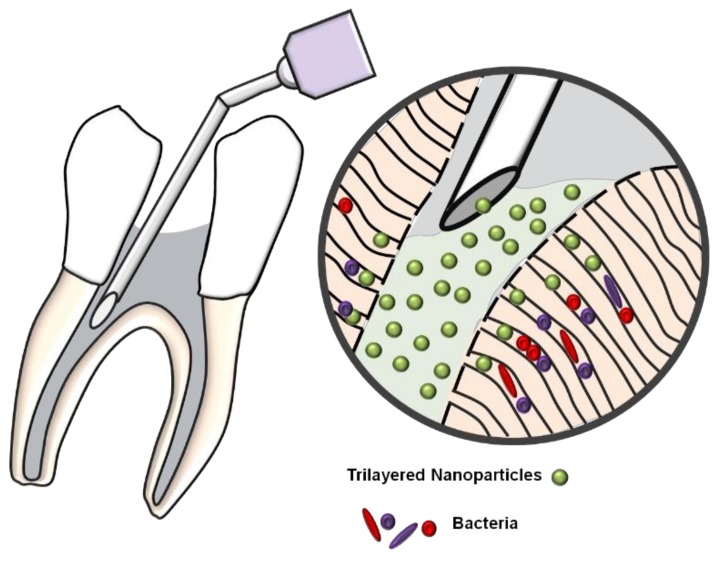
Schematics of the delivery of TNPs within dentinal tubules matrix.

The CHX-digluconate gel has been employed as an intracanal therapeutic drug. According to Gomes et al. (2013), it must remain inside the root canal system for at least 3 to 5 days [25]. Thus, it is important to provide sustained release of bactericidal for an extended period of time to improve root canal system disinfection. In vitro studies showed that 2% CHX gels may prevent *C. albicans* growth for 14 days, it may also diffuse through dentine and achieve the external root surface [26], and maintains its antimicrobial effect against *E. faecalis* for up to 12 weeks [27,28]. There is no consensus on the period that an intracanal medicament must remain inside the root canal system to achieve its maximum disinfection effect. However, it is important to develop alternatives to deliver the medicament and improve its diffusion through dentine tubules and the entire root canal system. To the best of the authors’ knowledge, this is the first time that a nanoparticle system composed of TNP encapsulated with CHX-digluconate is designed for targeted drug delivery in the root canal system (Figure 5).

The improved TNPs had a size range from 140–295 nm as determined by multiple characterization techniques. This is an important result because the goal was to obtain a size distribution that could penetrate the smallest dentinal tubules, for which size is reported to range from 500 nm to 1 μm [28]. The TNPs exhibited a steady CHX-digluconate profile and were suitable to be directly delivered deep into the complex matrix of dentinal tubules. Furthermore, investigation of the antimicrobial effectiveness of the CHX encapsulated TNPs experiments performed in bacterial broth demonstrated significant reduction in bacterial load up to 21 days, which supports the hypothesis that the system developed in this study will allow for continuous arrest of bacteria in a root canal anatomy. Earlier work by Haseeb and colleagues on BNPs demonstrated the effective encapsulation of CHX using the block copolymer PEG-*b*-PLA, which yielded nanoparticles with size range 300–500 nm in diameter [22]. On the other hand, the TNPs reproduced in this investigation were much smaller (140–295 nm) than their BNP counterparts, even with the same type of PEG-*b*-PLA block copolymer as starter material. A plausible explanation for the reduced size of the TNPs is the entangling of two critical polymeric chains, the PLA in the corona of the water-in-oil emulsion RNPs (emulsion-like loose nanoparticles), and the PLA chain that comes from the PEG-*b*-PLA block copolymer dissolved in an aqueous bulk solution [29]. This spontaneous self-assembly offers a moderately packed architecture because the hydrophobic polymeric chains of the corona (inner core) and the overlapping hydrophobic polymeric chains form a contracted shell. 

A common problem observed during purification of nanoparticles is clogging of filter pores. This is due to the presence of polyvinyl alcohol (PVA), a surfactant commonly used in the self-assembly of nanoparticles to improve their equilibrium in solution and reduce aggregation [30]. Thus, enhancing the self-assembly of TNPs in the reduction or total absence of PVA was a central task in this study. The optimal polymer to drug ratio to form the RNPs was found to be 4 to 1. This ratio was optimal to self-assemble the right polymeric shell of the TNPs without the use of PVA in the bulk solution, since PVA hinders an exertive filtration of nanoparticles by settling them at the pores resulting in a “filter cake” that obstructs the filter. This optimization enabled the purification step of TNPs without affecting the stability of the TNPs. This was apparent by the results of DLS spectra in which a high intensity peak was observed at 295 nm. The lower intensity peak at 105 nm may be related to BNPs, a byproduct of the self-assembly process. Moreover, these two peaks continued to display the same constant values even after leaving the final TNPs solutions at a standstill for 24 h.

The satisfactory drug entrapment efficiency (DEE) of CHX-digluconate (84.5%) can be accredited to the high concentration of PEG-*b*-PLA in the bulk aqueous solution to which the RNPs were added dropwise. Within this highly concentrated solution the polymer precipitated fast enough on the surface of the RNPs, which locked the drug inside the core of the TNP preventing diffusion across the phase boundary [31]. Furthermore, this effective encapsulation of CHX-digluconate may avoid premature precipitation of the insoluble CHX base in the oral cavity as it comes in contact with chlorine ions from saliva [23,32] or from residual sodium hypochlorite. In addition, this may render adequate transport of CHX into deeper areas of the dentinal tubular matrix, to arrest the bacterial growth of different microorganisms that can penetrate inside dentin at different depths [33].

The nanotoxicity study on *E. faecalis* revealed an early drug burst release from the TNPs. This event correlates to a reduced bacterial growth observed at day 1, which was probably associated to a fraction of the drug that was adhered or weakly attached onto the surface of TNPs. The second phase of CHX-digluconate release between 7 days to 21 days was attributed to diffusion mechanisms. This diffusion is directed by chemical potential gradients arising from osmotic pressure. In addition to diffusion, CHX-digluconate could be released by erosion of the polymer matrix, which leads to pore formation in the TNP [34]. The slow drug release between 7 days and 14 days declined bacterial proliferation by an additional 13%. Drug release between 14 days and 21 days declined bacterial proliferation an additional 17%, which resulted in a total accumulated bacterial inhibition of approximately 80%. The early drug burst release from TNPs was slower when compared with the BNPs that were previously studied [22]. Moreover, the second phase release of the TNPs had a steadier profile than the BNPs. The steadier release observed from TNPs could be attributed to their relative packed architecture from the overlapping of hydrophobic polymeric chains on the shell, as explained above.

In the earlier study involving BNPs, it was proved that when PEG-*b*-PLA nanoparticles were self-assembled without the addition of CHX and submerged in *E. faecalis* they did not have any bacterial inhibition. This experiment certified that the self-assembly of nanoparticles did not introduce reactive oxygen species (ROS) that could be toxic to cells [22]. ROS are partially reduced metabolites of oxygen, including hydrogen peroxide and hydroxyl radical, a by-product from polymer degradation during sonication of the emulsion [35,36]. Wang and collaborators have observed this degradation, which can start taking place after 5 to 15 min of sonication depending on the polymer [36]. ROS cause oxidative stress in many pathological pathways making them toxic to cells [37]. During sonication, sonolytic degradation of PEG-*b*-PLA nanoparticles was avoided by only sonicating for 4 min the RNPs dispersion. In this way, the nanotoxicity study of the TNPs discussed above can be interpreted based only on the activity of CHX-digluconate release. Thus, the TNPs can be classified as safe and suitable systems for delivery of CHX-digluconate inside the dentinal matrix.

In summary, this study developed and characterized a nanoparticle structure for efficient encapsulation of CHX for targeted drug delivery inside the anatomical dentinal matrix to be used in disinfection of the root canal. Some of the limitations of the study involved determining how far TNPs can penetrate throughout the different lengths dentinal tubules and what concentrations would cause too much adhesion of these TNPs to dentinal walls. A future study will discuss the design of a hydrogel matrix that would prevent TNPs from immediately adhering to the dentinal wall and aggregate. Additionally, experiments in vitro of the diffusion capabilities of these hydrogel-TNPs systems through the complex dentinal matrix may help clarify the actual depth of penetration and the fluid-flow mechanisms involved.

## 4. Materials and Methods

### 4.1. Materials

All chemicals were used as received from the manufacturers without any further purification, with the exception of tin(II) 2-ethylhexanoate, which was purified with high vacuum distillation. Poly(ethylene glycol) methyl ether [average molecular weight (MW) = 2000 g/mol], L-lactide, toluene, and pentane were all analytical grade and used to synthesize the block copolymer. Chlorhexidine digluconate (concentration 20% in H_2_O, density 1.06 g/mL), dichloromethane (DCM), 1,3-dimethyl-2-imidazolidinone (DMI), and N,N-dimethylacetoamide (DMA) were used to prepare the trilayered nanoparticles. All chemicals were obtained from Sigma-Aldrich (St. Louis, MO, USA). 

### 4.2. Polyethylene Glycol-block-Polylactic Acid (PEG-b-PLA) Synthesis

Poly(ethylene glycol)-*b*-poly(L-lactide) (PEG-*b*-PLA) was synthesized in house by ring-opening polymerization of L-lactide according to a previously reported method [38]. Briefly, 143 mg of poly(ethylene glycol) methyl ether (*M*_n_ = 2000 g/mol) and 1.03 g of L-lactide (Lactide-(3*S*)-*cis*-3,6-Dimethyl-1,4-dioxane-2,5-diene) were transferred to a Schlenk flask and stirred under vacuum for 1 h at 25 °C to remove moisture. Then the Schlenk flask was transferred to a nitrogen-filled box. 40 mg of tin(II) 2-ethylhexanoate (stannous octoate), as the catalyst in 0.5 mL of toluene, was added to the Schlenk flask. The flask was closed, taken out of the glove box, and heated in an oil bath at 110 °C overnight under continuous stirring to allow polymerization. The resulting polymer was precipitated in hexane and air-dried at room temperature to form a white powder.

The catalyst system delineated above was primarily chosen because of several advantages over other systems. Specifically, it is highly soluble in organic solvents and molten lactide in the bulk state, and it is stable during storage. The most significant advantage is that it is approved by the US Food and Drug Administration (FDA) for use in medical and food applications because it is biologically safe, although the mandate is dependent on empirical safety data [39].

### 4.3. Spectroscopy

^1^H NMR spectra were recorded on a Bruker ADVANCE III 500 MHz, (Bruker, Santa Barbara, CA, USA), in 5 mm sample tubes at 298 K (digital resolution of ±0.01 ppm) in DMSO-*d*_6_ using TMS as internal reference, with a spectrometer frequency for ^1^H at 500.23 MHz. The number-average molecular weight of each chain on the block copolymer was determined by ^1^H NMR. Sample temperature was kept constant for all measurements at 25 °C. The NMR peak of DMSO (δ = 2.54) was used as the reference in determining the chemical shifts of ^1^H in PEG-*b*-PLA. 

### 4.4. Reverse Nanoparticle Preparation

The first step to produce TNPs was to self-assemble RNPs. A water-in-oil emulsion was first prepared by sonicating for 4 min, in an ice bath. This emulsion was prepared as follows: a solution of 4 mL of 1,3-dimethyl-2-imidazolidinone (DMI) and N,N-dimethylacetoamide (DMA) mixed solvent (density adjusted to 1.00 g/cm^3^) was mixed with PEG-*b*-PLA block copolymer (4 mg) and 50 μL of aqueous solution (2 w/w %) containing chlorhexidine digluconate (10.6 mg/mL). The resulting water-in-oil emulsion was vigorously stirred for 2 h before measuring the diameter of the resulting RNPs. Then this emulsion was used in the self-assembling of TNPs.

### 4.5. Trilayered Nanoparticle Preparation

**Figure 6:**
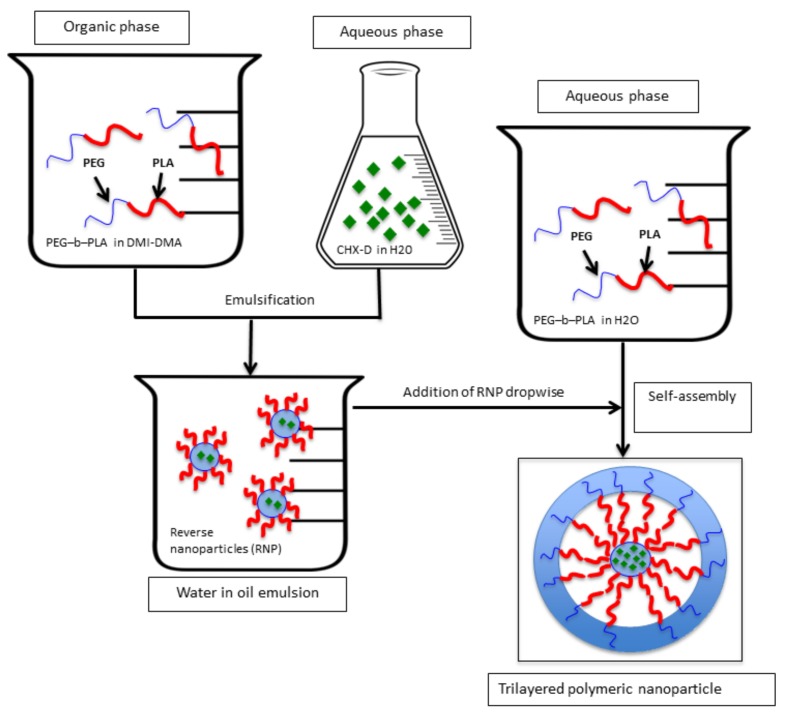
Schematic representation for assembling polymeric trilayered nanoparticles (TNP) with a hydrophilic core (chlorhexidine digluconate, a hydrophilic drug).

The scheme process for TNPs is shown in Figure 6. The water-in-oil emulsion recovered from the reverse nanoparticles (RNP) was added dropwise to 35 mL of aqueous solution containing PEG-*b*-PLA block copolymer (0.3 mg/mL). After 24 h of gentle stirring, the solution containing TNPs was dialyzed against deionized water by means of a SnakeSkin^TM^ Dialysis Tubing (molecular weight cut-off 3.5 KDa); Thermo Fisher Scientific Inc. Waltham, MA, USA). This step removed the residual organic solvents and unincorporated CHX digluconate.

### 4.6. Nanoparticle Size Measurements

Nanoparticle size is an important parameter, as chlorhexidine has to be directly delivered into the dentinal tubules with size ranging from 500 nm to 1.3 µm. A particle size less than 500 nm is then ideal to allow transport of the nanoparticles through even the smallest dentinal tubules. Dynamic Light Scattering (DLS) was employed to measure both particle size and particle size distribution using a Zetasizer Nano ZS90 (Malvern Instruments Ltd., Malvern, UK).

### 4.7. Dynamic Light Scattering

The prepared TNPs were dispersed in deionized water while the RNPs were dispersed in DMI-DMA mixed solvent (density adjusted to 1.00 g/cm^3^), then added to a quartz cuvette and analyzed by DLS (Zetasizer Nano ZS90, Malvern Instruments Ltd., Malvern, UK), to obtain their size distribution and polydispersity index (PDI). This experiment was replicated four times (*n* = 4) and each time the DLS frequency spectra was evaluated with the Zetasizer Nano ZS90 accompanying software.

### 4.8. Imaging

The evaluation of the TNPs’ morphology and size were analyzed with two different techniques: (1) scanning electron microscopy (SEM, ZEISS EVO^®^ LS-15, Cambridge, UK) using a variable pressure aperture, and (2) atomic force microscopy (AFM, Bruker, Bioscope Catalyst, Santa Barbara, CA, USA).

For SEM imaging, TNPs were first freeze dried then deposited onto a metallic 12-mm diameter stub. Copper sticky tape was used to reduce the build-up of electrons in the sample that can create unwanted image artifacts. The atomic material composition of the TNPs was measured with Energy Dispersive X-ray Spectroscopy (EDS, SEM-XRF, IXRF SYSTEMS Austin, TX, USA), which enabled detection of the individual elements of the nanoparticles.

For AFM analysis, CHX-encapsulated TNPs were dispersed in a hydrogel solution (1% Natrosol^TM^ hydroxyethyl cellulose in water). A drop of solution containing nanoparticles was deposited on a glass slide and was allowed to dry forming a thin film. AFM analysis was performed using PeakForce^TM^ quantitative nanomechanics (QNM) microscopy to map the morphology and quantitative measurement of material properties at a nanoscale level. For high-performance imaging and force control, specific probes, designed by Bruker SNL-10A, were selected. These probes had a spring constant of *k* = 0.35 N/m.

### 4.9. Drug Entrapment Efficiency Methodology

Drug entrapment efficiency (DEE) is defined as the percentage or fraction of the drug added in the process of nanoparticle synthesis that was successfully entrapped into the nanoparticle. The drug encapsulation efficiency (DEE) was calculated in relation to the theoretical drug loading (TDL), and the experimental drug loading (EDL) as described in Equations (1)–(3). The DEE of TNPs loaded with CHX-digluconate was calculated by constructing a calibration curve that relates CHX-digluconate wavelength at maximum absorbance to concentration. These absorbance measurements were collected using a UV-Vis spectrophotometry (Nanodrop 2000 spectrophotometer; Thermo Fisher Scientific, Waltham, MA, USA).

CHX-digluconate (Molecular Formula: C_22_H_30_Cl_2_N_10_·2C_6_H_12_O_7_; Molecular Weight: 897.8 g/mol) is one of the salt forms of CHX that a solubility in water up to 50% (w/v) and other solvents at room temperature. The creation of a calibration plot of absorbance versus concentration was successfully created due to the high solubility of CHX-digluconate. The first step was to obtain the highest absorbance of CHX-digluconate in dimethyl sulfoxide (DMSO) at different concentrations. Several solutions, which concentrations ranged from 0.064 mg/mL to 0.64 mg/mL, were prepared. Then a calibration curve was plotted using the maximum absorbance wavelength (λ_max_) and a linear fit calculated the absorbance as a function of CHX-digluconate concentration. The second step was to evaluate the assembled TNPs by dissolving them in 1 mL of DMSO then four different measurements (*n* = 4) of the absorbance at λ_max_ were taken to calculate the TNPs loading efficiency (Equations (1)–(3)). A PEG-*b*-PLA block copolymer curve was measured and evaluated to ensure that the polymer maximum absorbance (λ_max_) did not overlap with the one from CHX-digluconate.

(1) $$TDL=\;\frac{Weight\;of\;drug\;added\;\left(g\right)}{Weight\;of\;polymers\;and\;drug\;added\;\left(g\right)}\times 100\%$$

(2) $$EDL=\frac{Weight\;of\;drug\;after\;freeze\;drying\;\left(g\right)}{Weight\;of\;polymers\;and\;drug\;after\;freeze\;drying\;\left(g\right)}\times 100\%\;$$

(3) $$DEE=\;\frac{Experimental\;drug\;loading\;\left(EDL\right)}{Theoretical\;drug\;loading\;\left(TDL\right)}\times 100\%$$

### 4.10. Antimicrobial Effectiveness

Chlorhexidine-encapsulated TNPs were submerged in BHI (Brain Heart Infusion) broth at different time periods (1 day, 7 days, 14 days and 21 days). These time periods represent how long the nanoparticles would be dynamically active inside the complex dentinal matrix. The broth was then inoculated with *E. faecalis* at an initial optical density of 600 nm (OD600) of ~0.001.

The bacterial strain used in this study was the gram-positive bacterium *E. faecalis* (V583), which was isolated from the bloodstream of a hospitalized patient [40]. *E. faecalis* was selected for this proliferation assay because its pervasiveness in failed root canal treatments and chronic periodontitis [41].

First step: *E. faecalis* was cultured at 37 °C on 1.5% agar plates supplemented with BHI (Brain Heart Infusion) for 24 h. Second step: Preparation of the inocula for proliferation assays, an *E. faecalis* V583 colony from a freshly struck BHI agar plate was re-suspended in 10 mL. Step three: BHI broth was incubated for 24 h at 37 °C. Step four: 200 μL of the culture was aliquoted to inoculate the nanoparticles at the different time periods (1 day, 7 days, 14 days and 21 days). Step five: each TNP sample was inoculated in triplicate in a 96-well plate and incubated at 37 °C for 17 h. Three control trials were incubated in parallel as follows: Uninoculated BHI, BHI with TNPs and no *E. faecalis*, and BHI with *E. faecalis* and no TNPs. Step six: the OD600 was monitored with a Monochrometer-based Multi-Mode Microplate Reader (Synergy Mx, Winooksi, VT, USA) every 15 min during the 17 h incubation period. Step seven: The OD data was used to generate bacterial growth curves made by averaging triplicates from three trials together, not including clear outliers obtained during the experiments. The mean values of intermediate OD readings when the control broth was entering the growth phase were analyzed statistically with one-way ANOVA (analysis of variance) method at a 5% significance level. The OD readings of the broth that were inoculated with the TNPs were set side by side to the control sample to check if indeed the broth with CHX nanoparticles was the actual cause of delay in the bacterial growth phase.

## 5. Conclusions

The TNPs developed and investigated in this work presented a hydrophilic interior core that easily encapsulated CHX-digluconate. The TNPs had an adequate mean particle diameter to penetrate the anatomical structure of the dentinal matrix, thus presenting a potential methodology for local and sustained delivery of CHX. These also possessed good encapsulation efficiency, drug loading capacity, and showed steady inhibition of bacteria. The optimized TNPs had acceptable bioavailability against *E. faecalis*. There was an initial discharge of CHX-digluconate followed by a sustained release that was controlled by diffusion and degradation mechanism of the TNPs. These results established the potential application of TNPs for use in endodontic treatments as an intracanal regimen for better disinfection of the root canal system.

## Figures and Tables

**Table 1:** Energy dispersive X-ray spectroscopy (EDS) quantification analysis of selected element peaks.

Element	Intensity (counts/second)	Kα (KeV)	Concentration (weight %)
Carbon (C)	1910.74	0.277	37.24
Nitrogen (N)	13.61	0.392	1.44
Oxygen (O)	1884.44	0.525	60.05
Chlorine (Cl)	189.48	2.622	1.27
